# Ancient Metallurgy

**Published:** 1897-09

**Authors:** Henry Leffmann


### ANCIENT METALLURGY.[1]

BY HENRY LEFFMANN, M.D., D.D.S.[2]

ALTHOUGH it is the main object of this Society to discuss questions of actual dental practice, yet it has not been unwilling to turn its attention occasionally to theoretic subjects. The topic that I offer to-night is one that will not bear lengthy or frequent discussion, but it is not without interest, and to no specialists of medicine would it be so interesting as to dentists. Metallurgy forms a distinct feature of dental education. It is usually part of the title of some Faculty-chair in dental colleges, and its history cannot be entirely without interest.

Every one who has any knowledge of the history of chemistry knows that at one period the efforts of investigators were largely directed towards producing the precious metals from cheaper sources, and especially from the so-called base metals. As in so many other cases, however, accurate information as to the aims and methods of the alchemists is not wide-spread, and it was partly with a view of bringing some trustworthy data before you that I offered this paper. A very brief review of some general historic questions will be appropriate as an introduction.

The origin of chemistry is lost in the obscurity of antiquity. Even the derivation and significance of the name have been subjects of dispute. The Greek form "chemeia" is first noted in a

---

[1] Read before the Odontological Society of Pennsylvania, February 13, 1897.

[2] Professor of Chemistry and Metallurgy in the Pennsylvania College of Dental Surgery.

work of the fourth century of our era, although doubtless much older. It is now regarded as a Greek rendering of a name applied to Egypt; the word also means black, and various suggestions have been made concerning this. An eleventh century writer defined chemistry to be the art of preparing silver and gold.

Metallurgy must have been one of the earliest phases of chemic work. This would be expected *a priori*, and is shown by the allusions in the earliest records among various nations. The Hebrew Scriptures are regarded as containing references to six metals,—Au, Ag, Cu, Fe, Pb, and Sn. According to Pliny, the name metal (Gr. *met'alla*) is derived from the fact that they occur in association with others in veins, but other meanings are also given. The precious metals, gold and silver, which occur native, and have many striking properties, were in use at an early period, and there is evidence of the use of iron in Egypt in very early times. An incidental reference, showing the considerable antiquity of common metallurgic operations, is that in Proverbs xvii. 3: "The fining pot is for silver, and the furnace for gold: but the Lord trieth the hearts." The date is probably 500 B.C.

Positive evidence of the antiquity of the use of certain metals is obtained from the remains of ancient cities and temples. There has been so much successful digging during recent years that much of the hitherto-accepted ancient history has been overthrown, and it is unwise to express positive opinions as to the details of ancient civilization; but the following notes collected from some recent authorities, and seemingly trustworthy, may be offered:

Schliemann found in one of the layers in the mound at Hissarlik, supposed to be remains of Homeric Troy, articles of gold and silver, a bar of pure copper, and shapeless masses of lead. Leaden objects are uncommon among Egyptian remains, but Diodorus states that sheet-lead was used in the hanging gardens of Babylon. The oldest coined money known is that of Lydia; it was an alloy of gold and silver. The Greek and Roman bronze coinage from 500 B.C. to 50 B.C. contained from three to thirty per cent. of lead. This may have been put in to render the metal more fusible, but it may also have been a deliberate debasement by the government. References to lead occur in Homer and several other ancient writers. Mr. Eckfeldt, assayer at the United States mint at Philadelphia, has kindly given me a summary of the analyses of the Roman gold and silver coins in the mint collection. From this it appears that during the pagan period the coins were generally nearly pure metal.

In excavations at the site of the palace of Sargon, at Khorsabad, in 1854, votive tablets of different materials were found, to which a date of 700 B.C. is assigned. Four of these were examined by Berthelot; one is nearly pure gold, one nearly pure silver, one an alloy of tin and copper, much oxidized, and one magnesium carbonate. Another ancient article, a fragment of a vase, from a similar source, was found to be nearly pure antimony. This fact is quite remarkable, because pure antimony is even to-day very rarely employed, though its alloys are common. A votive figure from the same place was found to be pure copper, oxidized, however, to considerable depth.

These isolated instances are sufficient to show how early metallurgic operations began. Before passing to the consideration of alchemy, I wish to call attention to a feature of ancient metallurgy that has little to do with dentistry, but will be interesting because new to many. I refer to the use of lead pipes. This was quite familiar to the Romans. Tons of lead pipe have been dug up in various parts of Italy, and it has also been found in other parts of Europe that were under Roman domination. This pipe was made by folding sheets and securing the junction either by running melted lead along it or by the use of cement. Very often the name of the property-owner or of the plumber was imprinted on the sides of the pipe. Lanciani, the Italian archæologist, has described many of these inscriptions, and we learn, among other things, that women-plumbers were quite familiar to the ancient Romans. Such names as Julia Paula, Fabia Glycera, Aurelia Irene are among those given by Lanciani.

Vitruvius, a Roman writer on architecture, who lived about the beginning of the Christian era, refers to the danger of chronic lead-poisoning from water passing through lead pipes. I have a few lantern illustrations of Roman pipes and their applications.

Let us turn our attention to the special topic of alchemy.

Whatever may have been the aspirations and efforts of the ancient Greeks and Hebrews towards the transmutations of metals, they have left no definite evidence in their literature of efforts in such directions. The Jewish mind, in antiquity at least, was not scientific; and the Greek mind failed to grasp the true scientific method; in fact, the Greek philosophers disdained and condemned systematic observation and experiment. In later centuries, works were written and fraudulently ascribed to noted ancient personages, such as Moses, Democrites, Hermes, Solomon, Alexander, or Cleopatra. Indeed, it is very difficult to separate fact from fable and genuineness from fraud in this line of inquiry. As an example of this kind of apocryphal writing, I will refer to a paragraph in a manuscript of the eleventh century which is termed the "Diplosis of Moses,"—that is, a receipt originated by Moses for doubling the quantity of gold. A somewhat confused list is given, in which the words copper, sulphur, arsenic, lead, and sandarach are mentioned, though there is a little uncertainty in some of the terms; then a process of manipulation, which lasts for three days. We are then told that the added alloy will all be converted into gold, with, as the writer wisely adds, "the grace of God."

Within the past decade considerable accurate information concerning the alchemists has been brought into a readable form by the labors of the well-known French chemist, Berthelot. It had long been known that there are in some of the great European libraries Greek manuscripts on alchemy. Under the patronage of a department of the government of France, and assisted by various specialists in archæology and philology, Berthelot succeeded in sifting out much important matter from these writings and making *fac-similes* of some of the texts and illustrations.

The oldest of these manuscripts is now in the library at Leyden, in Holland. It is a papyrus, partly in Greek and partly in demotic character, found in a tomb at Thebes. It is believed to have been written in the third century of the Christian era, and is the oldest authentic manuscript of Egyptian alchemy. It is probably one of the few books that escaped the general destruction of books on alchemy and magic ordered by Diocletian about the close of the third century. It contains a long list of metallurgic receipts written in bad Greek, and is considered to be a sort of memorandum receipt-book of a metal-smith. The receipts show that a large part of the earlier alchemy was devoted to deliberate falsification. Manuscripts of later date, though still quite ancient, are also known. In these are found allusions to the relations between the planets and the known metals, also pictures of apparatus, and many mystical signs and obscure formulæ. I have had a few of these reproduced as lantern-slides, which will be shown later. The text of the oldest Leyden manuscript contains over one hundred receipts, from which a few may be taken as examples. Unfortunately, the translation is not always positive. Names that seem familiar and intelligible to us may not have the significance that we assign to them. Thus, magnesia is now understood to mean magnesium oxide, but in ancient writings it often means an iron oxide, magnetic oxide ($Fe_3O_4$), or even some other dark mineral. Sal ammoniac now refers to ammonium chloride, but originally it was applied to another substance not definitely understood. The term "chalk" is used with reference to a form of clay used as a flux.

There is some prominence given to receipts for the manufacture of an alloy called "*asem*," which is sometimes made of copper, tin, and mercury, sometimes of tin and mercury alone, sometimes more complex. Here are some translations from the receipts:

"*Manufacture of Asem.*—Tin, twelve drachms; mercury, four drachms; Chio earth (a sort of clay), two drachms. Add the powdered earth to the melted tin, then the mercury, agitate with an iron rod and allow to cool."

"*Another Receipt for Asem.*—Take tin in small and soft pieces, four times refined, four parts; pure white copper, three parts; asem, one part. Melt these and then purify the mass several times, and it is ready for use. It will be asem of the finest quality, which will deceive experts."

Here we have an evident intention to deceive by an alloy resembling silver.

Another receipt gives a method for augmenting gold,—namely, adding an alloy of copper and lead, with perhaps zinc, for some of the terms used are obscure.

You will all doubtless be interested in a receipt, written more than sixteen hundred years ago, for making gold-solder: "Gold, two parts; copper, one part; melt and granulate. If you wish a more brilliant color, add a little silver."

It is not necessary to multiply details. The text of these receipts shows a merely empiric metallurgy. Here and there we note a mystical trend, which becomes more marked in later writers. The ancient conception that all matter originates from one form contributed towards a belief in transmutation, and when motives of cupidity had led to the discovery of methods of imitating the precious metals by alloys which deceived even experts, it was no very great step to self-deception and the development of the futile search for the philosopher's stone.

An interesting feature of the early metallurgy was the supposed relation between the metals and the planets. We retain many traces of this to-day. Lead-poisoning is often called saturnine poisoning, silver nitrate is called *lunar caustic*, the preparations of iron are called martian preparations. By a chance, the number of moving stars visible to the naked eye is also the number of days in the quarterly period of the moon, and interesting usages have grown out of this coincidence, the discussion of which is not appropriate here. Various coincidences of a superficial character tended to connect the metals with the planets. The brightness of the sun accords with the rich brilliancy of gold; a very ancient allusion to this is in one of Pindar's odes. Similarly, the whiteness of silver corresponds to the paleness of the moon. The redness of Mars suggested iron, the bluish tint of Venus accorded with the lustre of the salts of copper. The dulness of the lustre of lead suggests a comparison with that of the planet Saturn. The assignments of Mercury, "the sparkling," and Jupiter, "the resplendent," have varied somewhat.

The list of relations between planets and metals has not always been as we now give it. In the earliest times certain alloys were regarded as distinct elements and classified as such. Thus, a natural alloy of gold and silver, called "electrum," and supposed to be a pure metal, was assigned to Jupiter. Tin was originally assigned to the planet Mercury, but later to Jupiter, when the metal, mercury, then called *hydrargyros*, was assigned to the innermost planet, to which its quick motion and sparkling lustre well suit it. Mercury was not known to the more ancient nations, but appears to have been discovered about 300 B.C. It was at first regarded as a sort of allotropic silver, if I may use a modern term, and represented by a crescent reversed, silver having been represented by the crescent upright. The notion that the native alloy of gold and silver, called electrum, is an element is found as late as the sixth century, in the work of Olympiodorus. Concerning this alloy there arose the not unnatural view that it could be made to yield either gold and silver at will. At the present day such a property would at once be regarded as proof of the existence of the two metals in the mass, but in earlier times such an explanation would not be suggested. The precipitation of copper from its solutions by the action of iron was regarded as the conversion of iron into copper; the small button of silver obtained by cupellation of lead-ores was regarded as a conversion of lead into silver. We know now that the silver is present in the original mass, but these ideas, which seem so simple and obvious to us, are matters of comparatively recent development.

The Egyptian alchemists regarded lead as the source of other metals, and as containing the transmuting agent; indeed, the term which we now translate lead originally covered any metal or alloy that was white and fusible. It will not be necessary to follow this phase of the subject further, and I pass to some brief allusions to forms of apparatus.

Enjoying as we do at the present time the benefit of instruments of precision and methods of inquiry by which opaque materials are open to our examination, we cannot appreciate the difficulties of manipulation under which the earlier workers labored. Even as late as the year 1777 we find Carl Wilhelm Scheele using pigs' bladders for collecting and studying the gases of the atmosphere.

The forms of apparatus that are figured in the alchemic manuscripts are principally those of distillation and crucible operations. Those that I will show on the screen are copies of Berthelot's *fac-similes* from a manuscript of the eleventh century now at Venice. There are inscriptions on various parts of the figures, and probably some of these were actually written on the apparatus, for these workers often mixed mysticism with practical ideas. Figures of similar apparatus are found in a fifteenth century manuscript now at Paris, and although there are some changes, yet we are still struck by the slowness with which progress was made when the lapse of four or five hundred years did not bring about complete abandonment of the older forms.

In regard to ancient metallurgic operations, it must be borne in mind that the ancients worked largely with mixtures of various ores, by which they obtained alloys at once, and, under the views then generally received, these alloys were regarded as single substances. We may smile at this: but when the nineteenth century began, chemists still regarded caustic soda as an elementary body. It will not be worth while to pursue this subject further, for it will become tedious. Indeed, I fear there has been already too much archæology and too little metallurgy. Before I pass to the lantern illustrations I wish to say a few words concerning the modern views as to the nature of alloys. Students often ask me if alloys are mixtures or compounds. If I pause to reflect before answering the question, the answers that float successively through my mind are: yes, no, both, neither, I do not know. The fact is that, until we can exactly define what we mean by mixture and combination, we cannot answer the question. We can, however, be sure that several different degrees of association exist in many alloys, and the probability is that most of them contain some of their constituent metals in a degree of association as close as we would expect in a combination, and this compound is mixed with the mass of the metal that is excess. For example, an alloy of ninety parts of silver and ten of copper probably consists of a compound of the two metals dissolved in— that is, mixed with—silver. It is, moreover, quite evident that the properties of many alloys are materially affected by slight impurities, among others, by the slight oxidation which almost all metals undergo when melted, and I think is an interesting line of research open to the one who will prepare alloys of absolutely pure ingredients.

We will now turn to the lantern illustrations.

